# Geometric and dosimetric uncertainties in intracranial stereotatctic treatments for multiple nonisocentric lesions

**DOI:** 10.1120/jacmp.v15i3.4668

**Published:** 2014-05-08

**Authors:** Brian Winey, Marc Bussiére

**Affiliations:** ^1^ Department of Radiation Oncology Massachusetts General Hospital and Harvard Medical School Boston MA USA

**Keywords:** patient setup, radiosurgery, dosimetric errors

## Abstract

The purpose of this study was to determine the effects of geometric uncertainties of patient position on treatments of multiple nonisocentric intracranial lesions. The average distance between lesions in patients with multiple targets was determined by a retrospective survey of patients with multiple lesions. Retrospective patient imaging data from fractionated stereotactic patients were used to calculate interfractional and intrafractional patient position uncertainty. Three different immobilization devices were included in the positioning study. The interfractional and intrafractional patient positioning error data were used to calculate the geometric offset of a lesion located at varying distances from the mechanical isocenter for treatments of multiple lesions with a single arc, assuming that no intrafractional position correction is employed during an arc rotation. Dosimetric effects were studied using two representative lesions of two sizes, 6 mm and 13 mm maximum dimensions, and prescribed to 20 Gy and 18 Gy, respectively. Distances between lesions ranged from < 10 mm to 150 mm, which would correspond to a range of isocenter to lesion separations of < 10 mm to 75 mm, assuming an isocenter located at the geometric mean. In the presence of a full six degree of freedom patient correction system, the effects of the intrafractional patient positioning uncertainties were less than 1.8 mm (3.6 mm) for 1σ (2σ) deviations for lesion spacing up to 75 mm assuming a quadratic summation of 1σ and 2σ. Without the benefit of a six DOF correction device, only correcting for three translations, the effects of the intrafractional patient positioning uncertainties were within 3.1 mm (7.2 mm) for 1σ (2σ) deviations for distances up to 75 mm. 1σ and 2σ deviations along all six axes were observed in 3.6% and 0.3%, respectively, of 974 fractions analyzed. Dosimetric effects for 2 mm and 4 mm offsets were most significant for the small lesion with minimum dose ($D_{\text{min}}$) decreasing from 20 Gy to 13.6 Gy and 5.7 Gy and volume receiving the prescription (V_20Gy_) reducing from 100% to 57% and 16%, respectively. The dosimetric effects on the larger lesion were less pronounced with $D_{\text{min}}$ reducing from 18 Gy to 17.5 Gy and 14.2 Gy, and V_18Gy_ reducing from 100% to 98.3% and 85.4%, for 2 mm and 4 mm offsets, respectively. In the 1σ scenario (3.6% of patients) angular uncertainties in patient positioning can introduce 1.0 mm shifts in the location of the lesion position at distances of 75 mm, compared to an isocentric treatment even with a full six DOF correction. Without the ability to correct angular positioning errors, a lesion positioned 75 mm away from the mechanical isocenter can be located in 3.6% of patients > 3.0 mm distant from the planned position. Dosimetric results depend upon the distance from isocenter and the size of the target. Single isocenter treatments for multiple lesions should be considered only when full six DOF corrections can be applied, the intrafractional immobilization precision is well quantified, and a PTV expansion is included for more distant lesions to account for unavoidable residual patient positioning uncertainties.

PACS number: 87.55.Qr, 87.53.Ly, 87.55.D‐

## I. INTRODUCTION

Recent studies have explored and demonstrated the use of volumetric‐modulated arc therapies (VMAT) for the treatment of multiple cranial lesions in a single arc.^1^, ^2^, ^3^, ^4^, ^5^, ^6^, ^7^, ^8^, ^9^, ^10^, ^11^ The primary benefit is the reduction of treatment time due to fewer isocenter setups, fewer imaging sequences, fewer treatment fields, and potentially fewer monitor units. The reduction in time has been suggested to increase the biological effectiveness of the dose^(3)^ and will undoubtedly increase the clinical efficiency and patient comfort.

When multiple lesions are treated with a single isocenter, the patient positioning uncertainty during the treatment will have different effects on the cumulative delivered dose compared to traditional isocentric treatment methods. Recent reports have analyzed the effects of rotational uncertainties on the delivered dose for isocentric lesions, but did not analyze lesions located at distances from isocenter.^12^, ^13^ Dose delivered to a lesion located at the mechanical isocenter of the treatment fields will be most affected by the linear translations of the patient position and less affected by small rotational uncertainties, unless the lesion has a particularly oblong shape.

The situation is potentially different when the lesion is located far from the mechanical isocenter of the treatment fields, as rotational patient uncertainties increasingly affect the accuracy of the location of the lesion. To date, none of the published reports detailing nonisocentric stereotactic treatments have thoroughly discussed the effects of rotational setup uncertainties, although some abstracts have been presented.^(14)^ Additionally, only two papers to date have presented full interfractional and intrafractional six degrees of freedom (DOF) data for their immobilization devices,^15^, ^16^ although some studies have measured intrafractional and laser‐based uncertainties in six DOF.^17^, ^18^ We explore the effects of these rotational uncertainties on the accuracy of lesion positioning and dosimetric consequences when treating a nonisocentric lesion.

## II. MATERIALS AND METHODS

### A. Intrafractional and interfractional patient positioning uncertainty

A retrospective analysis of fractionated stereotactic patient positioning data as ascertained from orthogonal imaging was conducted and previously described in detail, including the bite block immobilization (modified‐GTC, mGTC) specifications.^(16)^ Forty‐five random patients and 1002 fractions were analyzed with 974 intrafractional measurements. Orthogonal kV images were acquired prior to the treatment of the first treatment field of each fraction to correct for initial setup discrepancies, and image pairs were acquired prior to each treated field to correct intra‐fractional motion. The intrafractional and interfractional positioning data were obtained using a fiducial based 2D/3D back‐projection algorithm^(16)^ and are tabulated in Table 1.

**Table 1:** The intrafractional and interfractional accuracy and uncertainty of the mGTC frame^(16)^ and the VMP and UJS immobilizations.^(15)^

	Intrafraction			Interfraction		
*Dimension*	*mGTC*	*VMP*	*UJS*	*mGTC*	*VMP*	*UJS*
Lateral (LAT), mm	−0.12±0.37	−0.11±0.29	−0.11±0.28	−0.04±0.55	0.58±0.61	0.69±0.78
Ant/Post (AP), mm	−0.09±0.37	−0.03±0.21	0.01±0.26	0.09±1.29	0.40±0.48	0.46±0.78
Cran/Caudal (CC), mm	0.11±0.41	0.13±0.30	0.05±0.58	0.09±1.13	−0.47±0.95	−0.01±1.47
Pitch (about LAT), °	0.14±0.20	−0.02±0.14	−0.05±0.48	0.07±1.07	−0.42±0.38	−0.41±0.95
Yaw (about AP), °	0.10±0.50	0.06±0.27	0.02±0.46	−0.08±0.51	0.06±0.38	0.18±0.80
Roll (about CC), °	0.06±0.25	−0.02±0.15	0.02±0.49	0.05±0.59	−0.03±0.40	0.21±0.70

The distribution of intrafractional displacement was analyzed for all 974 intrafractional measurements to calculate the mean and standard deviations (SD) of the patient positioning uncertainty. Each fraction was also compared to the mean ± standard deviation for each of the six DOF to determine the displacement for each DOF. For all fractions analyzed, 3.6% of the fractions had displacements along all six DOF greater than 1 SD, and 0.3% had displacements along all six DOF greater than 2 SD.

As a comparison, a summary of a BrainLAB Novalis (Feldkirchen, Germany) system is also included in the Results section, which includes two separate immobilizations and full six DOF interfracational and intrafractional uncertainties of the respective devices as measured using the Novalis ExacTrac 2D/3D positioning system (BrainLAB AG).^(15)^ Forty patients and 1222 fractions were analyzed for interfractional motion and 400 fractions for intrafractional motion using the Novalis ExacTrac system prior to treatment and immediately following treatment. The ExacTrac positioning system employs a forward projection 2D/3D alignment system. The two immobilizations employed in the study^(15)^ are an upper jaw support (UJS) mask system and a vacuum mouth piece (VMP) frame with occipital support. Greater description of the immobilizations and methods can be found in the respective reports.

### B. Geometric uncertainty for nonisocentric targets

**Figure 1:**
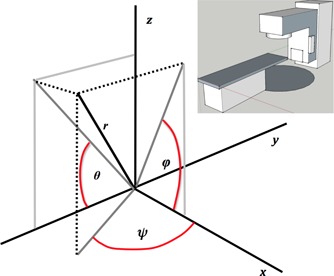
The coordinate system employed for this study. The distance r can vary as the distance between the mechanical isocenter (0,0,0) and the lesion center changes. E(r) is the magnitude of a shift in the location of lesion center (x, y, z). The insert provides a context of the coordinate system for a typical treatment couch.

For the calculation of the effect of patient positioning uncertainty at a distance, r, from the mechanical isocenter of the treatment field(s), we assume a simple geometric relationship detailed in Fig. 1, with the axes specified in Fig. 1. The position of the lesion center at distance r (mm) from isocenter can vary about the ideal position by a vector distance E(r) (mm). The definition of E(r) is separated into the angular (R) and linear (T) uncertainty components:

(1) $$R_{Pitch}(\mu_{\theta }+n\sigma_{\theta })=\left(\begin{array}{cccc} 1 & 0 & 0 & 0\\ 0 & \text{cos}(\mu_{\theta }+n\sigma_{\theta }) & -\text{sin}(\mu_{\theta }+n\sigma_{\theta }) & 0\\ 0 & \text{sin}(\mu_{\theta }+n\sigma_{\theta }) & \text{cos}(\mu_{\theta }+n\sigma_{\theta }) & 0\\ 0 & 0 & 0 & 1 \end{array}\right)$$

(2) $$R_{Roll}(\mu_{\theta }+n\sigma_{\theta })=\left(\begin{array}{cccc} \text{cos}(\mu_{\varphi }+n\sigma_{\varphi }) & 0 & \text{sin}(\mu_{\varphi }+n\sigma_{\varphi }) & 0\\ 0 & 1 & 0 & 0\\ -\text{sin}(\mu_{\theta }+n\sigma_{\varphi }) & 0 & \text{cos}(\mu_{\varphi }+n\sigma_{\varphi }) & 0\\ 0 & 0 & 0 & 1 \end{array}\right)$$

(3) $$R_{Yaw}(\mu_{\psi }+n\sigma_{\psi })=\left(\begin{array}{cccc} \text{cos}(\mu_{\psi }+n\sigma_{\psi }) & -\text{sin}(\mu_{\psi }+n\sigma_{\psi }) & 0 & 0\\ \text{sin}(\mu_{\psi }+n\sigma_{\psi }) & \text{cos}(\mu_{\psi }+n\sigma_{\psi }) & 0 & 0\\ 0 & 0 & 1 & 0\\ 0 & 0 & 0 & 1 \end{array}\right)$$

(4) $$T((\mu_{x}+n\sigma_{x}),(\mu_{y}+n\sigma_{y}),(\mu_{z}+n\sigma_{z}))=\left(\begin{array}{cccc} 1 & 0 & 0 & \left(\mu_{x}+n\sigma_{x}\right)\\ 0 & 1 & 0 & \left(\mu_{y}+n\sigma_{y}\right)\\ 0 & 0 & 1 & \left(\mu_{z}+n\sigma_{z}\right)\\ 0 & 0 & 0 & 1 \end{array}\right)$$

(5) r=(x y z 1)

(6) $$r^{\prime }=R_{Pitch}\;R_{Roll}\;R_{Yaw}\;\mathrm{T r}$$

and

(7) E(r)=‖r'−r‖

where *r* is the distance from mechanical isocenter to treatment target, and the six variables (μ_x_, μ_y_, μ_z_, μ_*th*_, μ_φ_, μ_Ψ_) represent the average setup positioning discrepancy of the respective variable, and σ_*t*_ is the standard deviation of the respective variable, t, as derived from the retrospective patient data analysis. The standard deviation was multiplied by factor n for values of 0, 1, and 2 which represents the average discrepancy and the average discrepancy plus 1 and 2 sigma deviations which incorporate 68% and 95% of the patient population for that variable. The summation of E(r) will occur much less frequently and was found in only 3.6% and 0.3% of all fractions treated for quadratic summation of 1 and 2 sigma uncertainty levels.

The summation of the mean and standard deviations describes the limits of the positional error value within the statistical model described by the mean and standard deviations. The resulting E(r) value is not an average positional value, but a limit achieved when the positional errors are aligned. E(r) was calculated for all combinations of (x,y,z) E {‐75,75}, *s.t.||r||* < 75 mm, and the maximum value of E(r) for each value of r was fit to generate a maximum curve for each scenario. The maximum error value of E(r) is a much less frequent occurrence in clinical situations, 3.6% and 0.3% of all cases for 1σ and 2σ limits. The order of the angular displacements was varied and the effect of the order of multiplication in Eq. (6) did not significantly affect the magnitude of E(r).

In Eq. (1), Θ, φ, and Ψ can be substituted with pitch, roll, and yaw in more clinical terms and x, y, and z can be substituted with Lat, CC, and AP directions.

### C. Translations versus rotations and translations

The analysis of patient positioning error was divided into two scenarios present in many radiation oncology centers: presence or nonpresence of rotational correction ability. For the scenario that a robotic couch or other rotational correction system is used for patient positioning and allows for rotational corrections, only the intrafractional rotational uncertainty is used in the positioning error calculations, E(r). In the case that a six DOF patient positioning system is not accessible for intracranial cases, the initial rotational uncertainty was added to intrafractional rotational uncertainty for the rotational component of the geometric uncertainty calculation, since this interfractional rotational error would not be corrected. For the translational component of the positioning error, interfractional translational patient positioning error was assumed to be accurately corrected such that only the translational intrafraction uncertainty was included in the E(r) calculations.

For both cases, six DOF and non‐six DOF, all positioning uncertainties are summed in order to project the total sum scenarios for the 1σ and 2σ cases. The uncertainties could fluctuate between ± and, therefore, the value of E(r) could be smaller than that displayed in the results. This point is particularly important to remember when the interfractional uncertainties are summed with the intrafractional uncertainties in the case of non‐six DOF correction.

### D. Separation of multiple lesions

A retrospective analysis of patient lesion locations was conducted. Thirty‐three patients with a total of 108 lesions (range 2‐17 lesions/patient) with multiple lesions treated in a single fraction during the last year at MGH were analyzed. The distances between all lesions, D (mm), were recorded and were assumed to be the Euclidean vector distance between the geometric centers of lesion 1 and lesion 2, as listed in the treatment planning system. Assuming that the mechanical isocenter is located at the center of the two lesions, the distance between lesions was divided by two in order to obtain a distance from isocenter to lesion center. For patients with more than two lesions, the distance calculation was repeated for all possible combinations of lesions to simulate the case that only two lesions would be treated in a single fraction.

### E. Dosimetric effects

A retrospective and representative planning study was conducted on two lesions to analyze the dosimetric consequences of a rotational intrafractional motion resulting in an error of patient positioning. The two lesions represented a smaller lesion (0.07 cc, 6 mm maximum dimension) and a larger, more typical size lesion (0.85 cc, 13 mm maximum dimension). Both lesions were treated in our clinic and were generally spherical. Six MV arc plans with cones were generated in CMS XiO (Elekta, Stockholm Sweden) to meet clinical prescriptions of 20 Gy to the smaller lesion and 18 Gy to the larger lesion with normalizations of 90% and no PTV margin expansion. The plans consisted of three arcs per lesion at roughly 45° couch kick separations and total arc degrees of 300.

Dosimetric effects were studied in the form of a DVH analysis for perfect alignment, a 2 mm AP displacement and a 4 mm AP displacement. The AP direction was chosen to represent an error dominated by the pitch of the patient position during treatment which is the most dominant error observed in our clinic. Positional errors of 2 and 4 mm were selected to capture the 1σ and 2σ maximum effects at a isocenter‐to‐lesion distance of 75 mm when a six DOF correction is applied.

## III. RESULTS

### A. Patient positioning uncertainty

A summary of the uncertainties of the mGTC immobilization device used in this report is listed in Table 1. As a comparison, the uncertainties from van Santvoort et al.^(15)^ can be found in Table 1 for two immobilizations, upper jaw support (UJS) and vacuum mouth piece (VMP). The mGTC and the UJS secure the upper jaw without vacuum assistance, whereas the VMP utilizes a vacuum assist device in the upper jaw immobilization.

For our data in Table 1, systematic errors of the alignment software and couch movement were also analyzed and were 0.1 mm/0.1° for translations and rotations. Hence, the intrafractional patient position uncertainty presented in Table 1 is mostly due to the residual patient motion in the immobilization device during the course of treatment. The averages of the displacements were within the systematic error.

### B. Geometric uncertainties: Case 6 DOF system

**Figure 2:**
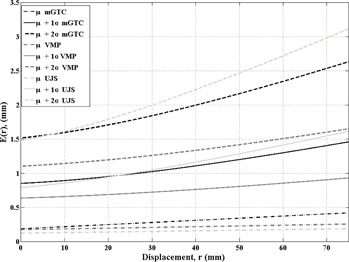
The net displacement, E(r), for distance, r is displayed for a full six DOF correction. The average plus 2 SD (dashed) represents the net displacement that 99.7% of patients would fall within (i.e., somewhere between average and average plus 2 deviations 99.7% of patients would reside). The black lines correspond to the mGTC immobilization, and the two shades of gray correspond to the data plotted for van Santvoort et al.^(15)^

Figure 2 details the net deviation of the lesion location as a function of the mechanical isocenter to lesion center distance. Three lines are displayed for the average displacement, average plus 0 SD, and average plus 2 SDs, for each of the three immobilizations, mGTC, UJS, and VMP, the last two from van Santvoort, et al.^(15)^ The black lines (solid, dashed, dash‐dotted) correspond to the mGTC frame of MGH. Table 2 provides a summary of the displacements at three discrete isocenter‐to‐lesion distances of 25, 50, and 75 mm.

**Table 2:** The magnitude of E(r) (in mm) for the intrafractional uncertainties at distances of 25, 50, and 75 mm for each of the three immobilizations for the scenario that a six DOF correction has been annlied

*r (mm)*	*Case*	*mGTC*	*VMP*	*UJS*
	μ	0.26	0.20	0.14
25	μ+1σ	0.98	0.70	0.99
	μ+2σ	1.77	1.22	1.89
	μ	0.34	0.23	0.16
50	μ+1σ	1.20	0.80	1.28
	μ+2σ	2.16	1.42	2.46
	μ	0.42	0.25	0.19
75	μ+1σ	1.46	0.93	1.61
	μ+2σ	2.63	1.65	3.11

At the zero separation, the net displacement is the baseline for the immobilization devices for isocentric treatments. The increase in displacement from the baseline is purely a function of the residual patient positioning uncertainty of the angular components of the immobilization devices. As can be seen from Fig. 2, all three immobilizations have similar translational accuracies and uncertainties, but the angular uncertainties are different, a difference that is magnified at distance from isocenter. The VMP device does have a smaller rotational uncertainty in general.

From the patient retrospective study, 3.6% of the patients had a displacement greater than the vector 1σ (i.e., the average vector displacement plus 1 SD line). The average plus 2 SD line represents the net displacement within which 99.7% of the patients would reside (i.e., 0.3% of the patients studied might have a vector displacement larger than the average plus 2 line). Along each of the six degrees of freedom, the data were Gaussian in distribution, but the summation of the errors is a random occurrence and only occurred in 3.6% and 0.3% of all patient fractions analyzed for 1σ and 2σ scenarios. In other words, only 0.3% of the fractions had positional errors along all six degrees of freedom greater than 2σ.

In Fig. 2 it can be seen that the angular uncertainty doubles the positioning uncertainty when approaching a distance of 75 mm (1.5‐3.1 mm). The difference of immobilization devices is also seen as the two immobilization devices without vacuum assist have larger angular uncertainties which correspond to large positional uncertainties at larger distances from isocenter.

Figure 2 also demonstrates the importance of a well‐quantified immobilization device for an individual institution as the VMP μ+2σ line intersects with the μ+1σ line for the UJS device at a distance of 75 mm.

### C. Geometric uncertainties: Case of no six DOF system

**Figure 3:**
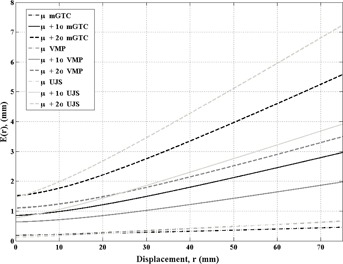
The net displacement, E(r), for distance, r is displayed for a scenario without a full six DOF correction. The average plus 2 SD (dashed) represents the net displacement that 99.7% of patients would fall within (i.e., somewhere between average and average plus 2 deviations 99.7% of patients would reside). The black lines correspond to the mGTC immobilization, and the two shades of gray correspond to the data plotted for van Santvoort et al.^(15)^

Figure 3 displays the effects of angular uncertainties when there is no angular correction capability in the patient positioning system. The baseline position (r = 0) is the same as in Fig. 2, but the slope of the lines are different as the angular interfractional uncertainties are added to the intrafractional angular uncertainties since the interfractional angular uncertainties are assumed to be uncorrected and, therefore, magnified during the treatment fraction.

Similar to the previous scenario, the VMP device μ+2σ line crosses the μ+1σ of the UJS device, but at a distance of 25 mm instead of 75 mm. Additionally, the range of lesion displacements increases to 7.2 mm at a distance of 75 mm for the 2σ scenario, which only occurs in 0. 3% of patient treatments.

### D. Lesion separation

**Figure 4:**
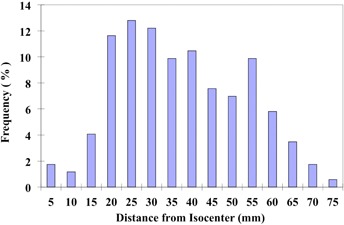
A summary of the displacements between lesions and isocenters in patients with multiple sites, assuming that the isocenter is equidistant from each lesion.

Figure 4 displays a histogram of the distances between lesions and assumed isocenters in patients with two targets. The range is < 5 mm to 75 mm assuming a mechanical isocenter location close to midpoint between lesions. Hence, the graphs in Figs. 2 and 3 cover the full range of distances observed in the institutions represented in this study. Thirty percent of the lesions (Fig. 4) were separated by a distance ≥ 100 mm or ≥ 50 mm separation from an isocenter located at the midpoint between two lesions.

### E. Dosimetric effects

**Figure 5:**
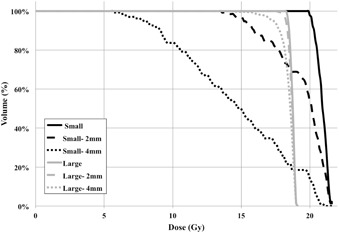
A DVH demonstrating the effects of 2 mm and 4 mm intrafractional patient position errors. The dosimetric impacts of small rotations when lesions are distantly located from the isocenter are most pronounced for the small lesion and diminished in the large lesion.

Figure 5 displays the dose‐volume histogram (DVH) curves for the two lesions and the three different isocenter positions (aligned, 2 mm AP error, and 4 mm AP error). The positional errors were selected to mimic the 1σ and 2σ errors of the mGTC at 50 mm distance from isocenter in the non‐six DOF scenario. Dosimetric effects were most significant for the small lesion with minimum dose ($D_{\text{min}}$) decreasing from 20 Gy to 13.6 Gy and 5.7 Gy and volume receiving the prescription (V_20Gy_) reducing from 100% to 57% and 16% for 2 mm and 4 mm offsets, respectively. The dosimetric effects on the larger lesion were less pronounced with $D_{\text{min}}$ reducing from 18 Gy to 17.5 Gy and 14.2 Gy and V_18Gy_ reducing from 100% to 98.3% and 85.4%, for 2 mm and 4 mm offsets, respectively. The effects were calculated assuming the lesion was offset for the entire treatment and clinical results would be dependent upon when the patient motion occurs during the fraction.

## IV. DISCUSSION

When treating nonisocentric lesions, the geometric uncertainty of the patient position becomes increasingly dependent upon the angular uncertainties of the immobilization and treatment positioning system. Proper quantification of an institution's patient positioning is required in order to accurately incorporate the effects of rotational uncertainties into the patient immobilization and treatment planning process.

The total uncertainty of the target location can be greater than 3.0 mm for 0.3% of patients for treatments of targets up to 75 mm from the mechanical isocenter for the mGTC frame and larger distances for the UJS and smaller distances for the VMP. Most patients will fall within the upper lines in Figs. 2 and 3, but adequate understanding of the uncertainty of the immobilization device is essential.

A distance‐ and lesion‐dependent PTV could be incorporated into the treatment planning process in order to account for the additional setup uncertainty encountered when treating with a nonisocentric treatment field. Again, the immobilization and institution specific uncertainties must be quantified for adequate PTV expansions.

For institutions that do not have an accurate means of a full six DOF patient setup correction, nonisocentric treatments would include greater difficulty as angular uncertainties for some immobilizations can have a larger effect on the lesion displacement at distance from mechanical isocenter when no angular correction is applied. Otherwise, a limit on the distance between the mechanical isocenter and the lesion center could be considered with adequate understanding of the immobilization uncertainty and a PTV expansion.

Several groups are exploring and utilizing surface imaging techniques that have the potential of reducing the intrafractional uncertainties that might contribute to positional variations when the lesion is distant from the mechanical isocenter.^19^, ^20^, ^21^

The advantages of a single‐arc VMAT treatment should not be diminished by this report. Uncertainties of patient positioning are unavoidable, but not impossible to overcome. Singlearc treatments for multiple isocenters might greatly reduce the time of treatment, which could contribute to less patient position uncertainty. This study did not correlate length of treatment with angular uncertainty.

Shorter treatments might also be more beneficial for patient comfort and palliative treatments when a patient might not otherwise be willing to undergo treatment.

Dosimetric impacts are an additional concern that cannot be generally addressed as they depend upon the patient, size and shape of the lesions, the planning system, the treatment planner, and mechanical specifications. We have presented two representative, nearly spherical lesions of two diameters. Size of the lesion greatly affects the overall dose distribution. Penumbras might cover some of the positional uncertainty, but the positional uncertainty must be quantified and understood in the planning process.

Finally, this report displays the summed error scenarios when the errors of patient setup are summed directly and for coverage of 96.4%‐99.7% of the patient population. Most patients will not have gross setup errors displayed by the μ.+1σ and μ.+2σ lines in Figs. 2 and 3, but the μ.+2σ lines are presented to display the effects in the most difficult patients. Since pretreatment imaging is routinely employed, a triage system could be employed for patients that display larger angular uncertainties (only isocentric treatments) and those who have small angular uncertainties (nonisocentric treatments).

## V. CONCLUSIONS

Residual angular uncertainties of patient positioning during intracranial treatments for treatment of nonisocentric lesions can add > 1 mm uncertainty in the location of the target with respect to the planned position when the distance between the isocenter and the lesion is greater than 75 mm in 3.6% of patients. Without six DOF positioning devices, the added positioning uncertainty increases to > 3 mm at 75 mm distance for 1σ scenarios. The patient immobilization device and setup uncertainties must be well determined in order to accurately model the dosimetric consequences. Proper modifications to the treatment planning process, particularly for small lesions, with the addition of a distance‐dependent PTV or limitations to the use of nonisocentric treatment applications of VMAT for intracranial targets should be considered.

## Supporting information

Supplementary Material
