# Supplementary figures and images for: Geometric and dosimetric uncertainties in intracranial stereotatctic treatments for multiple nonisocentric lesions
Source: J Appl Clin Med Phys. 2014 May 8;15(3):122–32. doi: 10.1120/jacmp.v15i3.4668 (PMC5711043; doi:10.1120/jacmp.v15i3.4668)

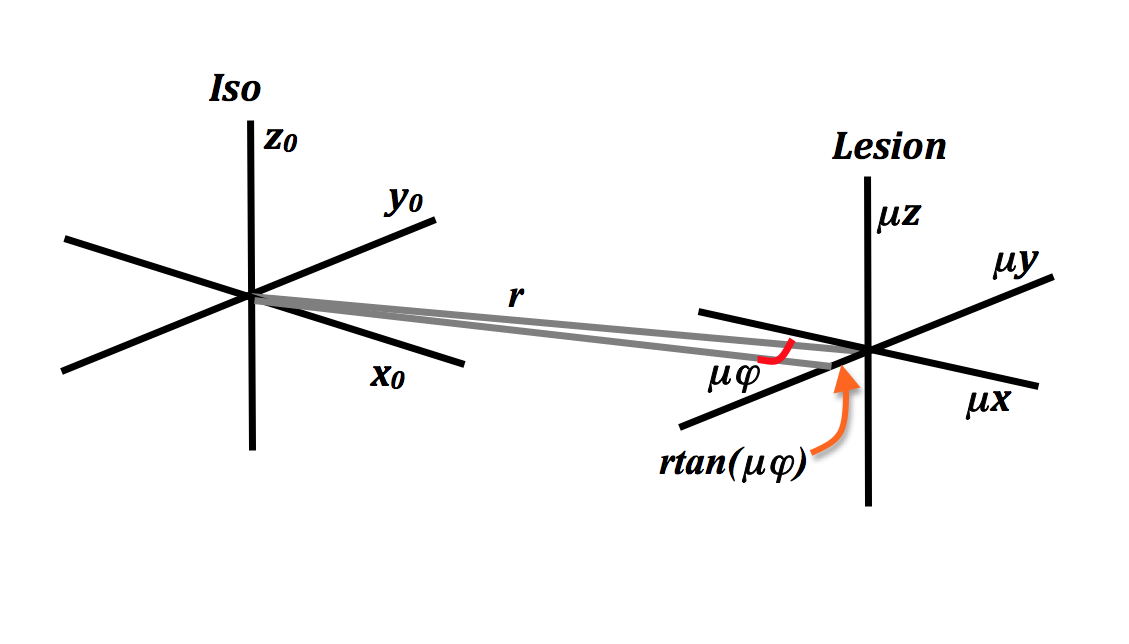

Supplement: Supplementary file 1 — Supplementary Material [file ACM2-15-122-s001.png]
